# Holliday junction recognition protein promotes pancreatic cancer growth and metastasis via modulation of the MDM2/p53 signaling

**DOI:** 10.1038/s41419-020-2595-9

**Published:** 2020-05-21

**Authors:** Chen-jing Wang, Xin Li, Ping Shi, Hai-yan Ding, Yan-ping Liu, Ting Li, Ping-ping Lin, Yun-shan Wang, Guo-qing Zhang, Yu Cao

**Affiliations:** 1 Department of Pharmacy, the Affiliated Hospital of Qingdao University, No.16, Jiangsu Road, Qingdao, Shandong 266003 China; 2 Department of Clinical Laboratory, The Second Hospital of Shandong University, 247 Beiyuan Street, Jinan, 250033 Shandong China

**Keywords:** Pancreatic cancer, Oncogenes

## Abstract

Holliday junction recognition protein (HJURP) refers to a histone H3 chaperone that has been implicated in different kinds of malignancies. Yet, its character in pancreatic cancer remains unclear. The expression of HJURP was assessed in PDAC tissues by RT-qPCR, immunoblotting, and immunohistochemistry. HJURP-deficient or overexpressed PDAC cell lines were constructed, using shRNA or plasmids with HJURP insert. MTT, sphere formation assay, migration, and invasion assays were performed to evaluate the viability, proliferation, migration, and invasion of PDAC cells. We used xenograft mice models to assess the tumor growth and metastasis in vivo. RNA-seq was applicated in search of the potential downstream target of HJURP in PDAC and subsequent verification were fulfilled via multiple assays, including immunofluorescence. Additionally, chromatin immunoprecipitation and luciferase reporter assay were conducted to explore the potential regulation of MDM2 expression by HJURP through H3K4me2. In this current research, we found that the expression of HJURP in PDAC cells and tissue was significantly higher than those of adjacent normal tissue, and high HJURP expression predicted poor survival. HJURP significantly promoted the viability, sphere formation, migration, and invasion of PDAC cells in vitro, HJURP also facilitated tumor growth and metastasis in vivo. Mechanically, MDM2/p53 axis is critical for HJURP-mediated malignant behaviors in PDAC, and HJURP regulates MDM2 expression through H3K4me2. HJURP could serve as a promising biomarker, and target for PDAC prognosis and treatment.

## Introduction

Pancreatic cancer is one of the most lethal malignancies, ranking seventh highest cause of cancer death worldwide^1^. In particular, pancreatic ductal adenocarcinoma (PDAC) is the most common malignancy of the pancreas, with an estimated 458,918 new cases diagnosed globally in 2018 and an associated 432,242 deaths in the same year^2^. The persistence of a dismal overall prognosis could be linked to a lack of sensitive and specific markers to help in pancreatic cancer early detection or currently undruggable genes with high mutation frequency, which results in limited therapeutic options^3^. Multiple genetic and epigenetic alterations, as well as the complex tumor microenvironments in PDAC, further challenge the medical treatment. Therefore, it is urgent to improve the understanding of the mechanism underlying the initiation and development of PDAC, so as to identify novel therapeutic targets that will improve the prognosis of PDAC patients.

Histone chaperones are proteins that escort histones throughout their cellular life, assisting histone folding, oligomerization, posttranslational modification, traffic, and nucleosome dynamics^4^. As indispensable regulators of chromatin structure and function, they are found to be frequently misregulated in cancer, which can have profound consequences on tumor growth and survival^5^. Holliday junction recognition protein (HJURP) is a histone H3 chaperone, responsible for CENP-A deposition at human centromeres during late mitosis/early G1 (refs. ^6,7^), which affects cell cycle progression in a CDK-dependent manner^8^. Like most other histone chaperones, the overexpression of HJURP has been observed in multiple cancers, such as bladder cancer, breast cancer, liver cancer, and lung cancer, as well as glioma^9–13^. However, HJURP’s function in PDAC remains unknown.

TP53 is one of the most common mutated genes in PDAC patients^3^, which encodes p53, a famous tumor suppressor protein interacting with MDM2 to mediate various biological processes. HJURP and CENP-A are known to transcriptionally upregulated in p53 null human tumors^14^. The previous study also demonstrated the role of HJURP in human fibroblasts and endothelial cells to regulate cellular senescence via a p53-dependent pathway^15^. Furthermore, the levels of PPARγ and acetylated-p53 were increased in the HJURP-deficient bladder cancer cells^9^. Pieces of evidence shown the potential relationship, and interaction between HJURP and p53, though, the underlying mechanism is still completely unknown.

The main purpose of this study was to detect HJURP’s expression in cell lines and tissue samples, assess the prognostic value of HJURP in PDAC patients, and illuminate the effect of HJURP on various cellular behaviors of PDAC cell lines both in vivo and in vitro. This current study also aimed to investigate potential signaling pathways and target proteins involved in the molecular mechanism underlying the regulatory effect of HJURP on the tumorigenesis and progression of PDAC.

## Materials and methods

### Clinical specimens

All of the PDAC and adjacent tissues were collected from 2012 to 2016 in the Affiliated Hospital of Qingdao University, China. This research was approved by the Ethical Review Committee of the Affiliated Hospital of Qingdao University and written informed consent was obtained from all the patients, who participated in the study.

### Cell culture

Human PDAC cell lines Capan-2 and SW 1990 were purchased from the ATCC (American Type Culture Collection). Cells were cultured in DMEM (Gibco, Grand Island, New York, USA) supplemented with 10% fetal bovine serum (FBS; Gibco), 1% penicillin and 1% streptomycin, and incubated in an incubator with 5% CO_2_ at 37 °C.

### RNA extraction and qRT-PCR

Total RNA was isolated from the PDAC and adjacent normal specimens, as well as the transfected PDAC cells, using TRIzol reagent (Invitrogen, Carlsbad, CA, USA). cDNA was obtained by using SuperScript II Reverse Transcriptase purchased from Invitrogen. The ABI PRISM 7900HT sequence detection system performed the qRT-PCR, and it has been involved in the data collection during the experiments.

### Western blot

Tissues lysates were electrophoresed on 10% SDS–PAGE gel and transferred to 0.45 μm PVDF membranes (Invitrogen, Carlsbad, CA, USA). The membranes were incubated with the primary antibodies overnight at 4 °C, including those for HJURP (1:1000), MDM2 (1:1000), p53 (1:2000), p21 (1:1000), Bax (1:1000), and Cyclin D1 (1:1000). The immunoblots were detected with a visual imaging system (Amersham Biosciences). β-actin was used as the loading control.

### Immunohistochemistry

Paraffin-embedded tissues were cut into 5 μm sections and incubated overnight at 4 °C, with the primary antibodies against HJURP, MDM2, and p53 (ABCAM, Cambridge, UK) at concentrations of 1:200.

### Cell viability assay

A total of 2000 cells/well were plated 12 h before treatment in 96-well plates. The cells were then handled for 72 h with specified agents. A total of 10% v/v of 5-mg/ml 3-(4,5-dimethylthiazol-2-yl)-2,5-diphenyltetrazolium bromide (MTT) solution was introduced for a duration of 2 h. The medium was then discarded and the cells dissolved in DMSO (Sigma, St Louis, MO). Relative cytotoxicity was determined by using a BMG Labtek plate reader to measure the absorbance at 570 nm. All expected experiments were conducted in triplicates and mean was calculated with SEM.

### Colony formation assay

For colony formation assessment, 2 × 10^3^ stably infected PDAC cells were seeded into six-well plates. After incubation for 15 days, the plates were washed with PBS three times and 4% paraformaldehyde was used to fix the cells for 25 min. Subsequently, the cells were stained with 0.5% crystal violet solution for further counting and statistical analysis.

### Immunofluorescence assay

For immunofluorescence assay, 5 × 10^4^ stably transfected CapanAPAN-2 cells were seeded in a 2 mm confocal plate for culture in the indicated incubator. Cultured cells were fixed with 4% paraformaldehyde and then permeabilized with 0.25% TritonX-100. After blocking with 1% bovine serum albumin, the primary antibodies against MDM2 (1:100) or p53 (1:100; company) were added into each plate for overnight incubation at 4 °C. The secondary antibodies (1:200, Sigma-Aldrich, USA) were incubated with the cells at 37 °C for 30 min, and DAPI was used to stain the nuclei at 37 °C for 10 min. The images were captured by a fluorescence microscope (Olympus BX53, Japan).

### Transwell assays

The 24-well transwell plates (Millipore, Bedford, MA, USA) with 8 μm pore filters were used for measuring cell migration and invasion. According to the manufactures’ instructions, 1 × 10^6^ PDAC cells per well were seeded in the serum-free medium into upper insert, with (invasion assay) or without (migration assay) Matrigel Matrix (BD, USA), and the subjacent compartment was incubated with medium, with 10% FBS at 37 °C in an incubator. After incubation for 48 h, PDAC cells migrating to the lower surface were stained with 0.5% crystal violet (Sigma-Aldrich, St. Louis, MO, USA) and photographed.

### RNA-seq analysis

For RNA-seq analysis, three batches of RNAs were isolated from cells using the PerfectPure RNA tissue kit. And 1 μg RNA was used to prepare libraries. Libraries were prepared using the Illumina TruSeq1 RNA Sample Preparation v2 according to manufacturer’s instructions. Indexed samples were sequenced on an Illumina HiSeq 2500 in a single read mode. The sequencing depth was 50×. The obtained reads, 50 bp long, were mapped to the genome assembly using TopHat2. Gene-level expression was quantified by applying HTSeq (version 0.6.1), and using the known genes from UCSC in gtf format as an annotation. Differential expression was calculated utilizing the DESeq2 software (version 1.2.10).

### ChIP assay

Chromatin Immunoprecipitation (ChIP) kit (cat. 17-371) was purchased from Millipore and ChIP experiments were carried out according to the manufactures’ instructions. Immunoprecipitated DNA was analyzed on the ABI PRISM 7900HT sequence detection system.

### Mouse xenograft assay

To assess the impact of HJURP on tumorigenesis and metastasis in vivo, 4-week-old male BALB/c nude mice. The mice were subcutaneously injected with 4 × 10^6^ PDAC cells per mouse, and sacrificed after 4 weeks for weight and volume measurements. To evaluate metastasis, the lungs of the nude mice were harvested for hematoxylin–eosin (H&E) staining and calculation of metastasis lesions after 10 weeks. All animal experiments were approved by the Ethics Committee for Laboratory Animals of the Affiliated Hospital of Qingdao University, China.

### Statistical analysis

The experiments were conducted in triplicates, and the data are presented as mean ± SD. Comparisons between groups were performed by Student’s two-tailed *t*-test. The overall survival rate curves of PDAC patients based on the Kaplan–Meier method were plotted using the log-rank test. *P* values <0.05 were considered statistically significant.

## Results

### HJURP is highly expressed in PDAC cells and tissues

**Fig. 1:**
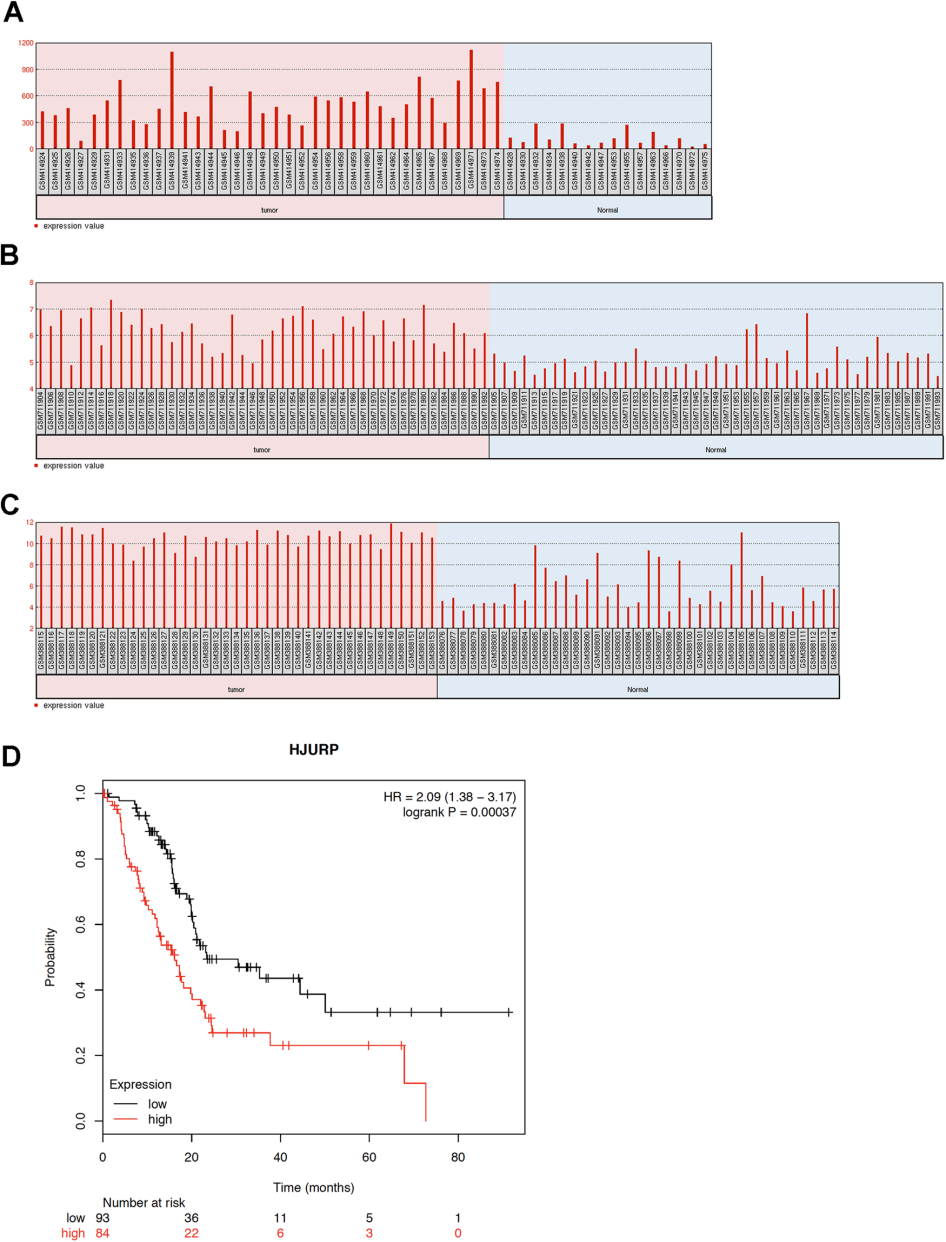
Expression of HJURP is upregulated in human PDACs and related to poor clinical outcome. **a**–**c** Gene expression of HJURP in human PDACs compared to normal tissues from three GEO datasets. **d** Survival analysis of PDAC patients with high (red; *n* = 84) or low (black; *n* = 93) HJURP expressions.

Our analysis with data from three Gene Expression Omnibus (GEO) datasets (GSE16515, GSE28735, and GSE15471) showed increased HJURP expression in PDACs compared to normal tissues samples (Fig. 1a–c). Additionally, we noted that patients with high HJURP levels had significantly poor survival compared to those with low HJURP levels (*p* < 0.001; Fig. 1d).

**Fig. 2:**
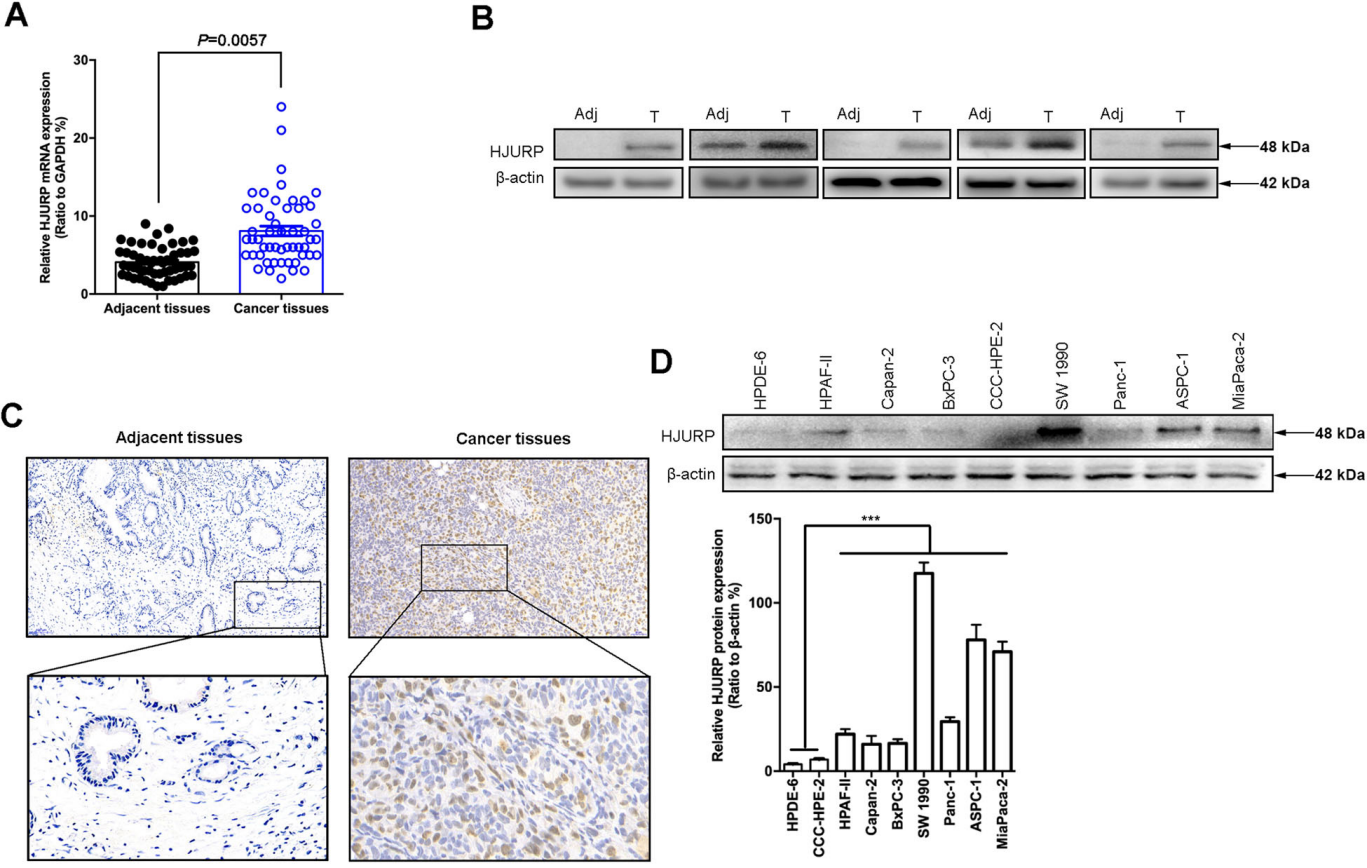
HJURP is overexpressed in PDAC specimens and cell lines. **a** mRNA expression of HJURP in 219 clinical PDAC samples and matched adjacent normal tumor samples. **b**, **c** Representative images of immunoblotting analysis (**b**) and immunohistochemical (IHC) staining (**c**) of HJURP in PDACs compared to normal tissues. **d** Protein level of HJURP in PDAC cell lines and normal pancreatic cells. All experiments were repeated at least three times, and representative data are shown. Scale bars, 50 μm (**c**) in the upperand 20 μm in the lower (**c**); data are means ± SEM; **p < 0.01.

We next detected the mRNA levels of HJURP in PDAC specimens and adjacent normal tissues by using RT-qPCR, immunoblotting, and immunohistochemistry (IHC) analysis (Fig. 2a–c). Consistently, high HJURP expression was observed in cancer tissues compared to normal tissues. Besides, we examined protein levels by immunoblotting in PDAC cell lines and found that HJURP was also apparently upregulated (Fig. 2d). In addition, we also analyzed the ROC curves of HJURP in pancreatic cancer patients and healthy controls (AUC = 0.753, *p* < 0.05; Supplementary Fig. 1). Taken together, the above-mentioned results indicate that HJURP might be correlated with PDAC progression.

### HJURP promotes PDAC tumor growth both in vitro and in vivo

To examine this hypothesis, PDAC cell lines Capan-2 and SW 1990 were transfected with lentiviral vectors encoding human HJURP inserts or shRNAs according to the endogenous expression of HJURP. The transfection efficiency was confirmed by immunofluorescence of GFP, immunoblotting, and RT-qPCR (Supplementary Fig. 2).

**Fig. 3:**
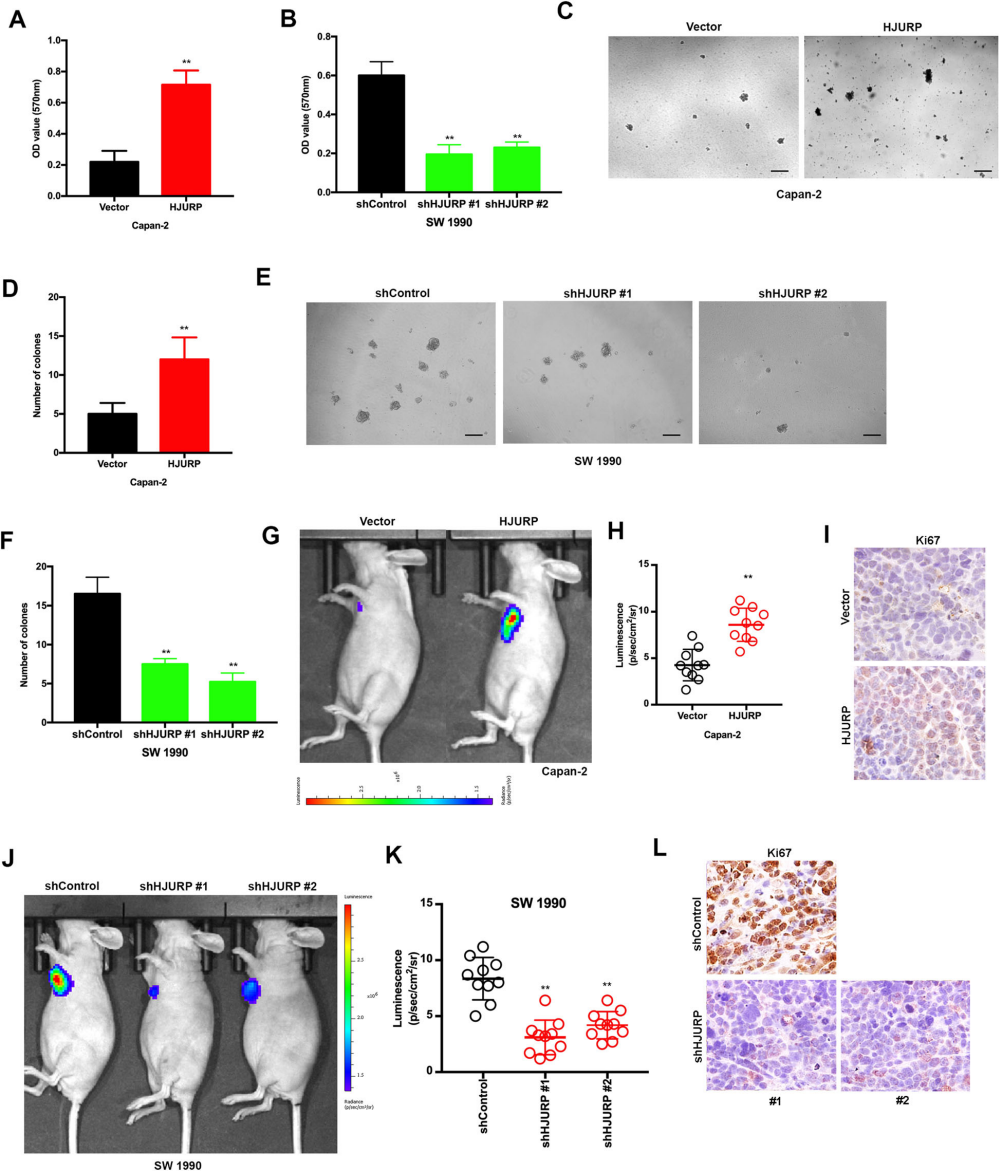
HJURP promotes PDAC growth in vitro and in vivo. **a**, **b** MTT assays in HJURP overexpression (**a**) and knockdown (**b**) PDAC cells. Capan-2 (**a**) and SW 1990 (**b**) cells were transfected with HJURP or shHJURP vectors, respectively. **c**–**f** Pancreatosphere formation (**c**, **e**) and its quantification (**d**, **f**) of PDAC cells mentioned in **a** and **b**. **g**–**l** Representative ventral view images (**g**, **j**) and its quantification (**h**, **k**) of bioluminescence from xenograft mice transplanted with cells described above. The representative images, IHC analysis of Ki-67-positive cells of mouse allograft tumors from Capan-2 (**i**) and SW 1990 (**l**; *n* = 5). All experiments were repeated at least three times, and representative data are shown. Scale bars, 100 μm (**c**, **e**) and 20 μm (**i**, **l**); data are means ± SEM; **p < 0.01.

Subsequently, cell viability and sphere formation assays were performed to detect the impact of HJURP on tumor proliferation. As expected, the ectopic expression of HJURP promoted both cell viability and sphere formation in Capan-2 cells (Fig. 3a, c, d). Conversely, the knockdown of HJURP dramatically inhibited those oncogenic behaviors in SW 1990 cells (Fig. 3b, e, f).

In agreement with these in vitro findings, xenografts injected with Capan-2 cells bearing HJURP inserts displayed exceeding tumor formation compared to control models (Fig. 3g–i), whereas deficiency of HJURP in the SW 1990 cells significantly inhibited the tumor growth in vivo (Fig. 3j–l). Collectively, the above-mentioned observations indicate that HJURP promotes PDAC tumorigenesis both in vitro and in vivo.

### HJURP contributes to migration, invasion, and metastasis in PDAC

**Fig. 4:**
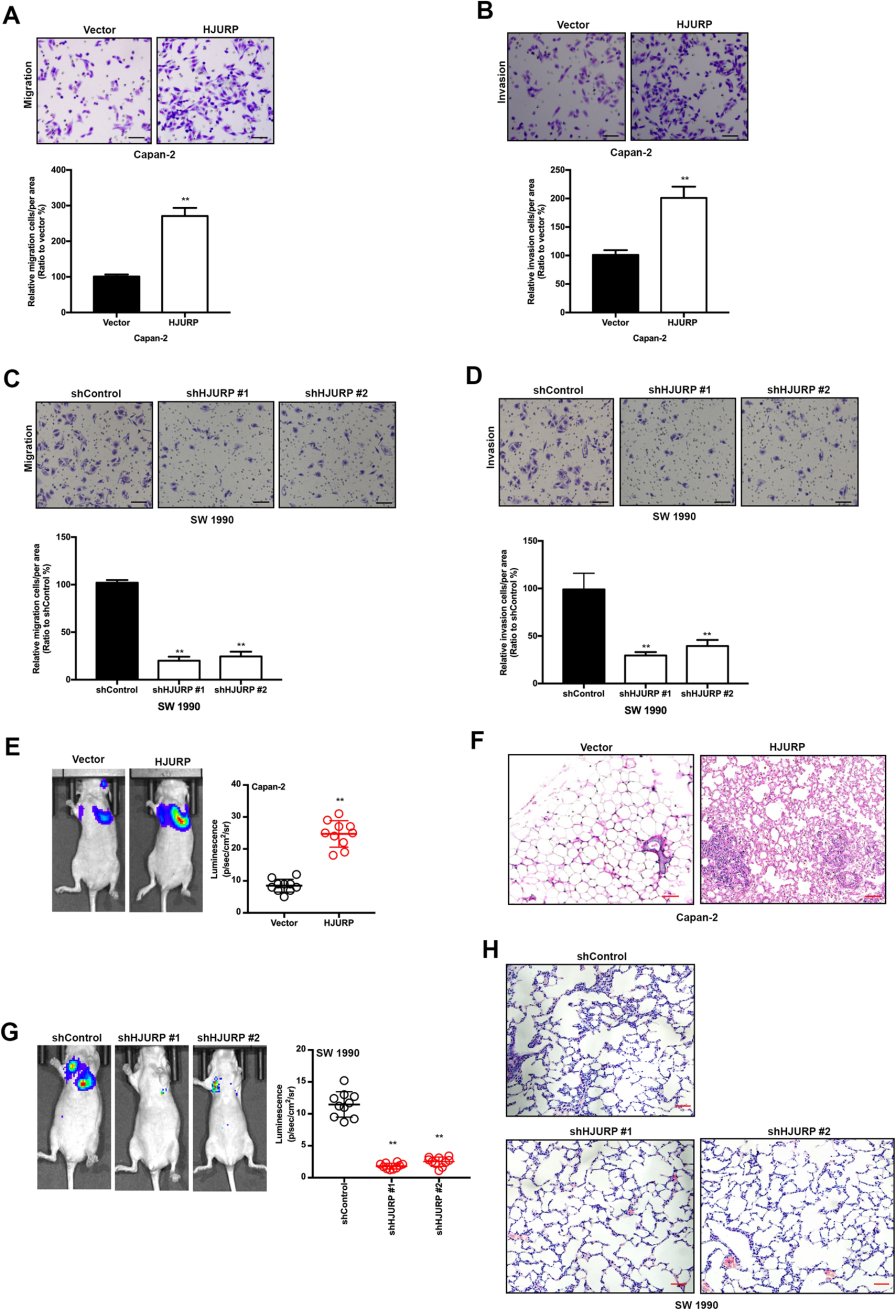
HJURP promotes PDAC migration, invasion, and metastasis in vitro and in vivo. **a**–**d** Migration (**a**, **c**) and invasion (**b**, **d**) of transformed Capan-2 (**a**, **b**) and SW 1990 (**c**, **d**) cells described in Fig. 3 (top), with quantification (bottom). **e**–**h** Representative ventral view images of bioluminescence (**e**, **g**) and hematoxylin–eosin staining of lung sections (**f**, **h**) from migration mice. All experiments were repeated at least three times, and representative data are shown. Scale bars, 20 μm (**a**–**d**) and 50 μm (**f**, **h**); data are means ± SEM; **p < 0.01.

HJURP has been implicated in cell proliferation, migration, and invasion via mediating Wnt/β-catenin signaling in non-small cell lung cancer (NSCLC)^16^. To further determine whether the HJURP-induced malignant behavior of PDAC cells, Capan-2 cells with excessive expression of HJURP and SW 1990 cells, with HJURP depletion were subjected to cell migration and invasion assessment. As expected, HJURP overexpression enhanced migration and invasion of PDAC cells (Fig. 4a, b), whereas HJURP knockdown reversed the effects (Fig. 4c, d), suggesting that HJURP facilitated cell migration and invasion in PDAC.

Consistent with in vitro observations, tail vein injected with Capan-2 cells bearing HJURP vectors displayed enhanced lung metastasis compared to control models (Fig. 4e, f). In contrast, a drastic reduction in metastasis was observed in the tail vein injected with HJURP-deficient SW 1990 cells (Fig. 4g, h). These results thus indicate that HJURP assists migration and invasion of PDAC cells, consequently promoting in vivo metastasis.

### HJURP affects MDM2/p53 axis in PDAC

**Fig. 5:**
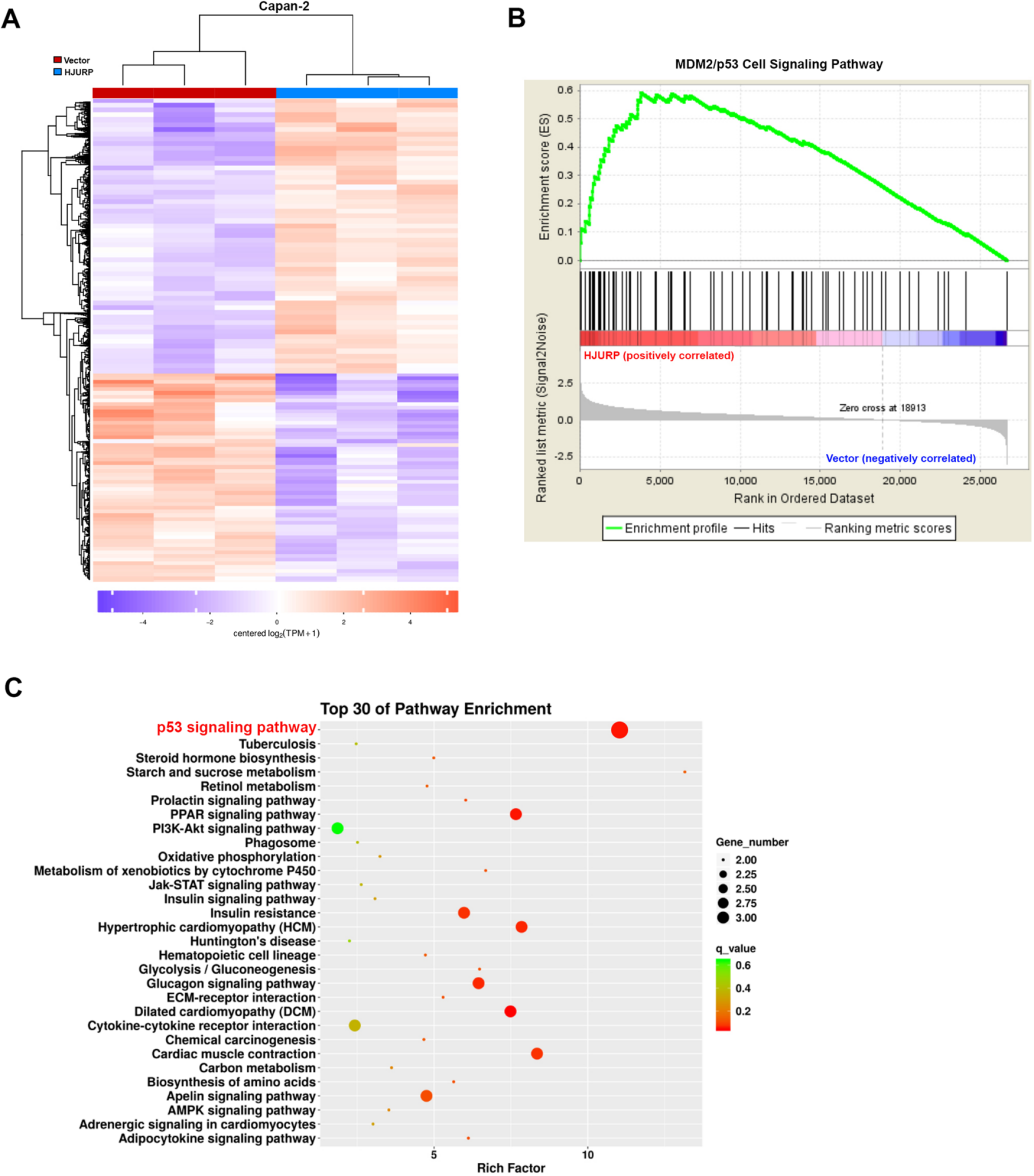
HJURP depletion inactivates multiple signaling pathways. **a** Heatmap summarizing genes differentially expressed in Capan-2 cells transfected with HJURP or empty vectors. **b** Volcano plot displaying differentially expressed genes. Upregulated genes (3123) are highlighted in red. Downregulated genes (2477) are highlighted in green. Black dots represent genes not differentially expressed. **c** Enrichment of an MDM2/p53 cell signaling pathway in GSEA analysis of genes altered as described above.

To decipher the underlying mechanism of PDAC development and progression driven by HJURP, we performed RNA-seq with Capan-2 cells bearing empty vectors or HJURP inserts (Fig. 5a). As shown in the volcano plot, a total of 3123 genes was found positively associated with HJURP overexpression, while 2477 genes are negatively correlated (Fig. 5b). Gene set enrichment analysis (GSEA) revealed the enriched signature related to endogenous HJURP-dependent transcription in PDAC, namely, the MDM2/p53 signaling pathway (Fig. 5c).

**Fig. 6:**
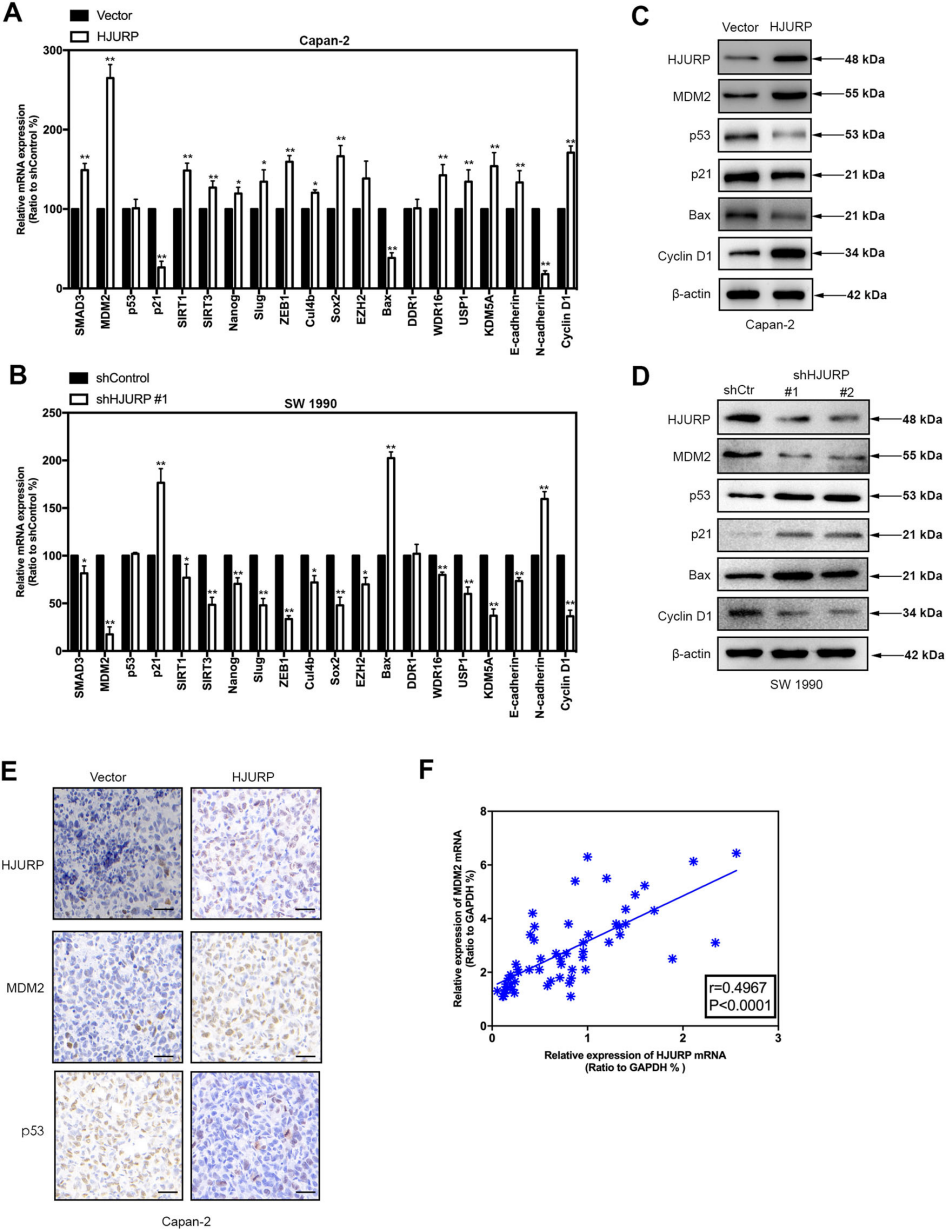
HJURP mediates the MDM2/p53 axis. **a**, **b** mRNA expression of the top 20 genes significantly differentially expressed in transformed Capan-2 (**a**) and SW 1990 (**b**) cells described in Fig. 3. **c**, **d** Immunoblotting of proteins involved in MDM2/p53 signaling pathways with transformed Capan-2 (**c**) and SW 1990 (**d**) cells as described above. **e**, **f** Representative images of IHC staining (**e**) and immunofluorescence (**f**) of MDM2/p53 in transformed Capan-2 as described above. The positive relationship between mRNA expression of HJURP and MDM2 in pancreatic cancer tissues. Scale bars, 20 μm (**e**).

MDM2 is known to be a primary cellular inhibitor of p53, which induces p53 degradation and inactivates its tumor-suppressing activity. p53 is one of the most well-studied tumor suppressor proteins and regulates downstream genes that are involved in DNA repair, cell cycle arrest, or apoptosis^17^. To explore HJURP’s impact on the MDM2/p53 axis, RT-qPCR and immunoblotting were applied to total RNA and protein extracted from Capan-2 and SW 1990 cells (Fig. 6a–d). Apparently, HJURP overexpression upregulates MDM2 expression at both mRNA and protein levels in Capan-2 cells, contrary to the effect of HJURP depletion in SW 1990 cells. By comparison, p53 is only affected at the protein level by HJURP alteration, suggesting HJURP may mediate p53 in an indirect way, for instance, via MDM2. Further confirmative evidence came from the IHC and immunofluorescence experiments on Capan-2 cells, which again demonstrates the positive relationship between HJURP and MDM2, as well as the negative relationship between HJURP and p53 (Fig. 6e, f, Supplementary Figs. 3 and 4). Besides, RT-qPCR analysis of human PDAC tissues also validated the positive relationship between HJURP and MDM2 at the mRNA level (Fig. 6g). To further verify that HJURP negatively regulates p53 through MDM, Capan-2 cells overexpressing HJURP were treated with an MDM inhibitor (Nutlin-3a), and the results showed that MDM inhibitors reversed the negative regulation of HJURP on p53 (Supplementary Fig. 5a). These results show the effect of HJURP on p53 expression levels through MDM.

Taken as a whole, these observations suggest that HJURP probably modulates p53 signaling pathways through mediating MDM2 expression, which is known to degrade p53 via ubiquitination.

### HJURP regulates MDM2 expression through H3K4me2

**Fig. 7:**
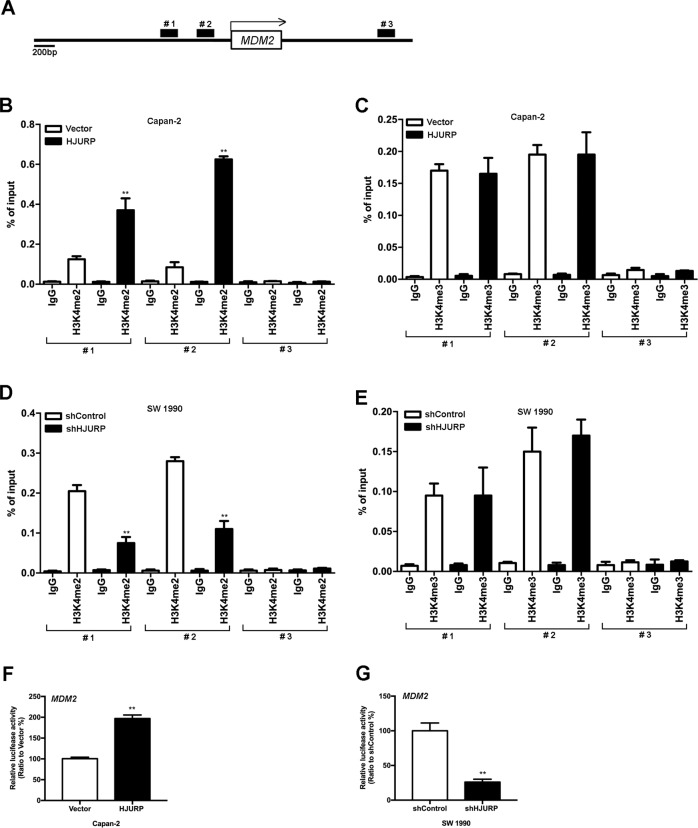
HJURP increases the binding of dimethylation of H3K4me2 at the promoter region of MDM2 and upregulates its transcription. **a** A schematic representation of the promoter region of MDM2. **b**–**e** Transformed Capan-2 (**b**, **c**) and SW 1990 (**e**, **f**) as described above were chromatin immunoprecipitated for IgG and H3K4me2 (**b**, **d**) or tri-methylation of lysine 4 on histone H3 (H3K4me3; **c**, **e**). Pull down at the three MDM2 promoters (**e**) was assessed by RT- qPCR, and calculated as the percent of IgG input. Error bars are SEM for three technical replicates. **f**, **g** Luciferase reporter assays showing the impact of overexpression (**f**) or deficiency (**g**) of HJURP on MDM2 promoters in Capan-2 (**f**) or SW 1990 (**g**) cells. All experiments were repeated at least three times, and representative data are shown; data are means ± SEM; **p < 0.01.

Epigenetic engineering studies have shown that dimethylation of histone H3 at lysine 4 (H3K4me2) is required for HJURP targeting and CENP-A assembly on a synthetic human kinetochore^18^. To figure out whether this is the case, three possible binding sites for H3K4me2 were then predicted on the promoter region of MDM2 (Fig. 7a). Accordingly, Capan-2 cells bearing HJURP or control vectors were chromatin immunoprecipitated (ChIP) for IgG and H3K4me2, respectively, followed by qPCR (Fig. 7b). As expected, there is a significant increase of H3K4me2 binding at two putative binding sites (#1 and #2) in Capan-2 cells bearing HJURP inserts compared to control cells. However, no significant change was observed for binding of tri-methylation of lysine 4 on histone H3 (H3K4me3) at any of the putative binding sites in Capan-2 cells, with or without HJURP overexpression (Fig. 7c), suggesting the specificity of H3K4me2 binding on MDM2 promoter regulated by HJURP. In comparison, a significant decrease of H3K4me2 binding at the two putative binding sites (#1 and #2) was identified in SW 1990 cells transfected with shHJURP compared to control cells, whereas no such difference was found for binding of H3K4me3 (Fig. 7d, e). These observations were further confirmed by luciferase reporter assay that ectopic expression of HJURP transcriptionally activated the promoter of MDM2, whereas depletion of HJURP inactivated the promoter (Fig. 7f, g). Additionally, histone methyltransferase inhibitor, GSK343, was used to treat Capan-2 cells overexpressing HJURP. The experimental results showed that GSK343 significantly inhibited the level of H3K4me2 and reversed the upregulation of MDM expression level by HJURP (Supplementary Fig. 5b). These data together provide compelling evidence that HJURP promotes the binding of H3K4me2 at the promoter region of MDM2 to activate its transcription.

### MDM2 is critical for HJURP-mediated malignant behaviors of PDAC cells

**Fig. 8:**
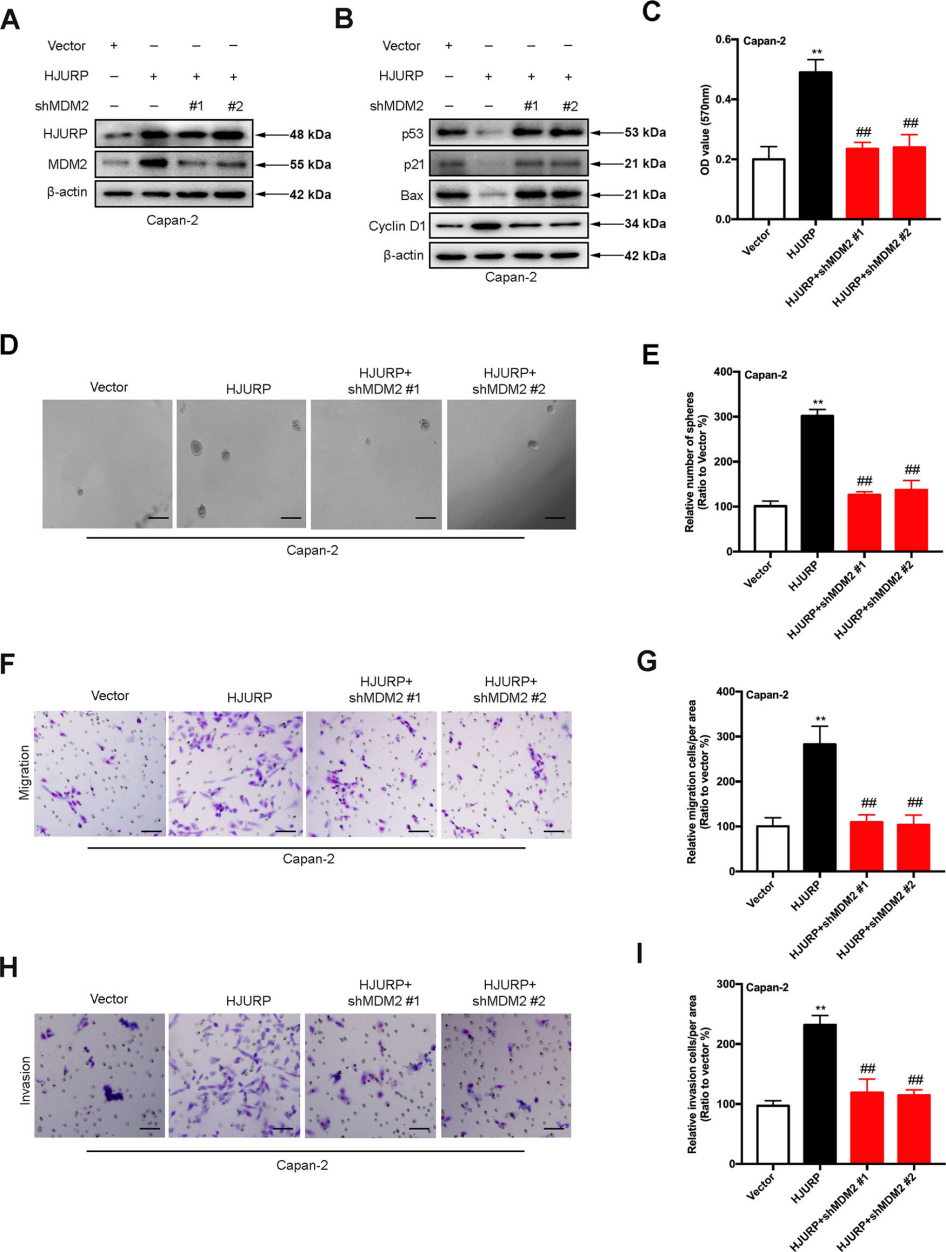
MDM2 is required for HJURP-induced malignant behaviors in PDAC. **a**, **b** Immunoblotting of HJURP, MDM2 (**a**), and proteins involved in p53 signaling pathways (**b**) with Capan-2 cells expressing HJURP insert as well shRNAs that target MDM2. (**c**–**i**) MTT (**c**), sphere formation (**d**) and its quantification (**e**), migration (**f**) and its quantification (**g**), and invasion (**h**) and its quantification (**i**) of BXPC-3 cells described above. All experiments were repeated at least three times, and representative data are shown. Scale bars, 100 μm (**d**) and 20 μm (**f**, **h**); data are means ± SEM; **p < 0.01.

To prove the involvement of MDM2 in HJURP-induced malignancy, we established stable Capan-2 cell lines expressing HJURP insert as well shRNAs that target MDM2. As anticipated, MDM2 knockdown completely abolished the effect of HJURP overexpression on p53 and its downstream targets, including p21, Bax, and Cyclin D1 (Fig. 8a, b). Similarly, MTT and sphere formation assay showed that MDM2 deficiency dramatically reduced the impact of HJURP overexpression on cell viability and sphere formation (Fig. 8c, d, e). Besides, it was found that the promoting effect of HJURP on cell migration and invasion was blocked after MDM2 knockdown (Fig. 8f–i), indicating that HJURP might facilitate the malignant behavior of PDAC cells via the MDM2/p53 signaling pathway.

## Discussion

The incidence of PDAC is increasing yearly and 355,317 new cases are estimated to occur until 2040. Despite improvements in the detection and treatment, the 5-year survival rate of pancreatic cancer is only 9% (ref. ^1^). Certain risk factors like smoking, diabetes, and obesity, have been identified; however, a better understanding of the etiology and identification of crucial risk factors are still essential for the primary prevention of this disease.

HJURP has been demonstrated to be correlated with multiple human neoplasms, including bladder, breast, liver, and lung cancer, as well as glioma^9–13^. However, HJURP’s role in PDAC remains elusive. In this study, we observed that HJURP was significantly overexpressed both in PDAC cell lines and clinical specimen. Additionally, Kaplan–Meier analysis showed that high HJURP expression was correlated with shorter overall survival in PDAC patients. These results suggest that HJURP probably exerts an oncogenic role in PDAC, which has not been reported before.

Although several studies have referred to HJURP’s abnormal existence in tumors, little is known about its biological function and mechanism in tumors. According to current studies, HJURP acts as a tumor promoter in HCC by mediating cell proliferation, migration, and invasion both in vitro and in vivo^11,19,20^. A similar effect was also observed in NSCLC as HJURP deficiency inhibits NSCLC cell proliferation, migration, and invasion via repressing Wnt/β-catenin signaling. However, the underlying mechanism of HJURP-regulated PDAC tumorigenicity has not been elucidated. In this study, to explore the specific impacts of HJURP on PDAC behaviors, we used si-HJURP to downregulate the levels of HJURP in SW 1990 cells, and HJURP inserts to overexpress the protein in Capan-2 cells. Subsequently, a batch of phenotypic assays was performed to investigate the role of HJURP. As a result, HJURP knockdown suppressed colony formation, migration, and invasion of PDAC cells, whereas HJURP upregulation showed the opposite effect. The in vitro results were further confirmed in xenograft mice models. Thus, we revealed that HJURP promotes PDAC cell proliferation, migration, and invasion in vitro, and facilitates tumor growth and metastasis in vivo.

The MDM2/p53 signaling pathway, a highly conserved molecular mechanism, plays a critical role in the regulation of DNA repair, cell cycle arrest, or apoptosis. This pathway has been involved in the modulation of various tumors via mediating its downstream targets, such as p21, Bax, Cyclin D1, and so on. Aggregating evidence has shown the potential relationship and interaction between HJURP and p53, though, the underlying mechanism is still insufficiently known. In this study, we performed RNA-seq with Capan-2 cells bearing empty vectors, or HJURP inserts to explore the downstream target of HJURP and MDM2/p53 signaling stood out in the subsequent GSEA enrichment. Further western blotting, IHC, and immunofluorescence experiments confirmed the impact of HJURP on the MDM2/p53 axis. mRNA analysis showed that HJURP modulated MDM2 expression instead of p53, suggesting HJURP-regulated p53 and its downstream targets via mediating MDM2. Most importantly, MDM2 deficiency remarkably undermined the impact of HJURP overexpression on cell viability, sphere formation, cell migration, and invasion, further demonstrating that HJURP might assist the malignant behavior of PDAC cells via mediating the MDM2/p53 signaling pathway. The above mechanism was more suitable for pancreatic cancer in p53 wt. And in pancreatic cancer with p53 mutation or null, how does HJURP promote tumorigenesis? Since the experimental results of p53 null were not involved in our research, we make a guess: first, when p53 was mutated or null, the expression level of HJURP is significantly increased. Therefore, mutation or deletion of p53 does not affect the regulation of MDM2 expression by HJURP. We found that overexpression of HJURP caused an increase in MDM2 expression, which also affected p21, Bax, and Cyclin D1 protein levels. Therefore, in p53 mutations or null, HJURP may increase pancreatic cancer development through MDM2 regulation of other tumor suppressor molecules (such as p21, Bax, and Cyclin D1). In p53 mutant or null pancreatic cancer, the function and mechanism of HJURP will be the focus of our next research.

H3K4 methylation is the modification at the fourth lysine residue from the N-terminus of histone H3, including mono-methylation (H3K4me1), dimethylation (H3K4me2), or tri-methylation (H3K4me3), which is related to the transcriptional activation of genes^21^. It has been reported that H3K4me2 depletion prevented kinetochores from efficiently recruiting HJURP (ref. ^18^), suggesting the essential role of H3K4me2 for HJURP’s potential assembly. However, it is not known if H3K4me2 is also required for HJURP’s function. ChIP-qPCR assays were thus performed with transformed Capan-2 or SW 1990 cells to explore the impact of HJURP on the binding of H3K4me2 to MDM2 promoter. Together with luciferase reporter assay, the results demonstrate that HJURP activates MDM2 transcription via regulating the recruitment of H3K4me2 at its promoter region. In order to further understand the more specific regulatory mechanism of HJURP on the MDM promoter region, we will continue to explore transcription factors that may be combined with the MDM promoter (# 1 and # 2). Therefore, finding possible transcription factors will be the focus of our recent verification.

The expression of HJURP in PDAC cells and tissue was significantly higher than that in adjacent normal tissue, and high HJURP expression predicted poor survival. HJURP significantly promoted the viability, sphere formation, migration, and invasion of PDAC cells in vitro, and facilitated tumor growth and metastasis in vivo. Mechanically, the MDM2/p53 axis is critical for HJURP-mediated malignant behaviors in PDAC and HJURP regulates MDM2 expression through H3K4me2. HJURP could thus serve as a promising biomarker and target for PDAC prognosis and treatment.

## Supplementary information

Supplemental Fig. 1

Supplemental Fig. 2

Supplemental Fig. 3

Supplemental Fig. 4

Supplemental Fig. 5

Supplemental Figure legends

## References

[1] Rawla P, Sunkara T, Gaduputi V (2019). Epidemiology of pancreatic cancer: global trends, etiology and risk factors. World J. Oncol..

[2] Bray F (2018). Global cancer statistics 2018: GLOBOCAN estimates of incidence and mortality worldwide for 36 cancers in 185 countries. CA Cancer J. Clin..

[3] Kleeff J (2016). Pancreatic cancer. Nat. Rev. Dis. Prim..

[4] Hammond CM, Stromme CB, Huang H, Patel DJ, Groth A (2017). Histone chaperone networks shaping chromatin function. Nat. Rev. Mol. Cell Biol..

[5] Nye J, Melters DP, Dalal Y (2018). The Art of War: harnessing the epigenome against cancer. F1000Res.

[6] Dunleavy EM (2009). HJURP is a cell-cycle-dependent maintenance and deposition factor of CENP-A at centromeres. Cell.

[7] Shuaib M, Ouararhni K, Dimitrov S, Hamiche A (2010). HJURP binds CENP-A via a highly conserved N-terminal domain and mediates its deposition at centromeres. Proc. Natl Acad. Sci. USA.

[8] Stankovic A (2017). A dual inhibitory mechanism sufficient to maintain cell-cycle-restricted CENP-A assembly. Mol. Cell.

[9] Cao R (2017). Silencing of HJURP induces dysregulation of cell cycle and ROS metabolism in bladder cancer cells via PPARgamma-SIRT1 feedback loop. J. Cancer.

[10] Montes de Oca R (2015). The histone chaperone HJURP is a new independent prognostic marker for luminal A breast carcinoma. Mol. Oncol..

[11] Hu B (2017). Holliday junction-recognizing protein promotes cell proliferation and correlates with unfavorable clinical outcome of hepatocellular carcinoma. Onco Targets Ther..

[12] Zhou D, Tang W, Liu X, An HX, Zhang Y (2017). Clinical verification of plasma messenger RNA as novel noninvasive biomarker identified through bioinformatics analysis for lung cancer. Oncotarget.

[13] de Tayrac M (2013). Prognostic significance of EDN/RB, HJURP, p60/CAF-1 and PDLI4, four new markers in high-grade gliomas. PLoS ONE.

[14] Filipescu D (2017). Essential role for centromeric factors following p53 loss and oncogenic transformation. Genes Dev..

[15] Heo JI, Cho JH, Kim JR (2013). HJURP regulates cellular senescence in human fibroblasts and endothelial cells via a p53-dependent pathway. J. Gerontol. A Biol. Sci. Med. Sci..

[16] Wei Y (2019). Knockdown of HJURP inhibits non-small cell lung cancer cell proliferation, migration, and invasion by repressing Wnt/beta-catenin signaling. Eur. Rev. Med. Pharm. Sci..

[17] Hientz K, Mohr A, Bhakta-Guha D, Efferth T (2017). The role of p53 in cancer drug resistance and targeted chemotherapy. Oncotarget.

[18] Bergmann JH (2011). Epigenetic engineering shows H3K4me2 is required for HJURP targeting and CENP-A assembly on a synthetic human kinetochore. EMBO J..

[19] Chen T (2018). HJURP promotes hepatocellular carcinoma proliferation by destabilizing p21 via the MAPK/ERK1/2 and AKT/GSK3beta signaling pathways. J. Exp. Clin. Cancer Res..

[20] Chen T (2019). HJURP promotes epithelial-to-mesenchymal transition via upregulating SPHK1 in hepatocellular carcinoma. Int. J. Biol. Sci..

[21] Li S, Shen L, Chen KN (2018). Association between H3K4 methylation and cancer prognosis: a meta-analysis. Thorac. Cancer.

