# Supplementary figures and images for: Holliday junction recognition protein promotes pancreatic cancer growth and metastasis via modulation of the MDM2/p53 signaling
Source: Cell Death Dis. 2020 May 21;11(5):386. doi: 10.1038/s41419-020-2595-9 (PMC7242411; doi:10.1038/s41419-020-2595-9)

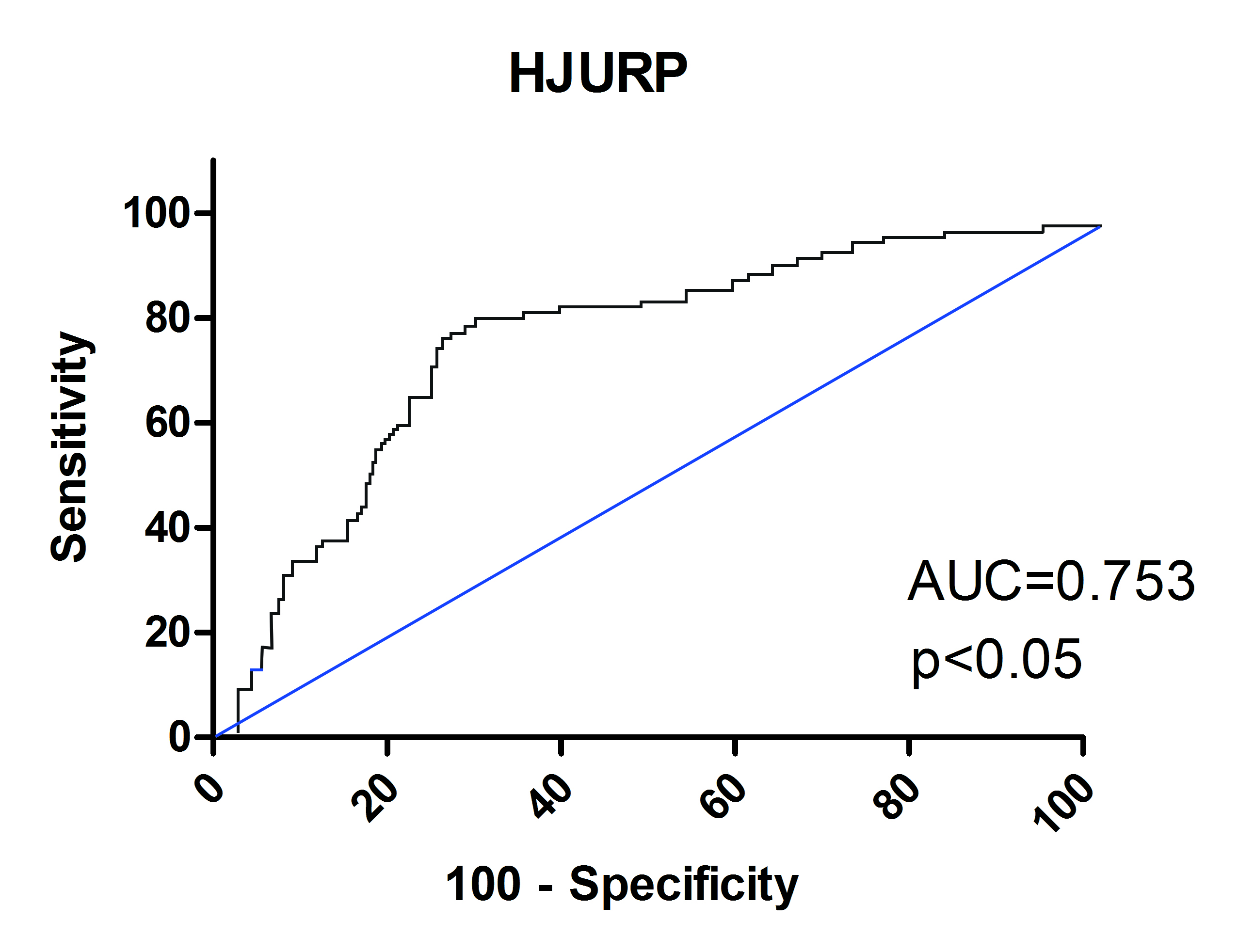

Supplement: Supplementary file 1 — Supplemental Fig. 1 [file 41419_2020_2595_MOESM1_ESM.tif]

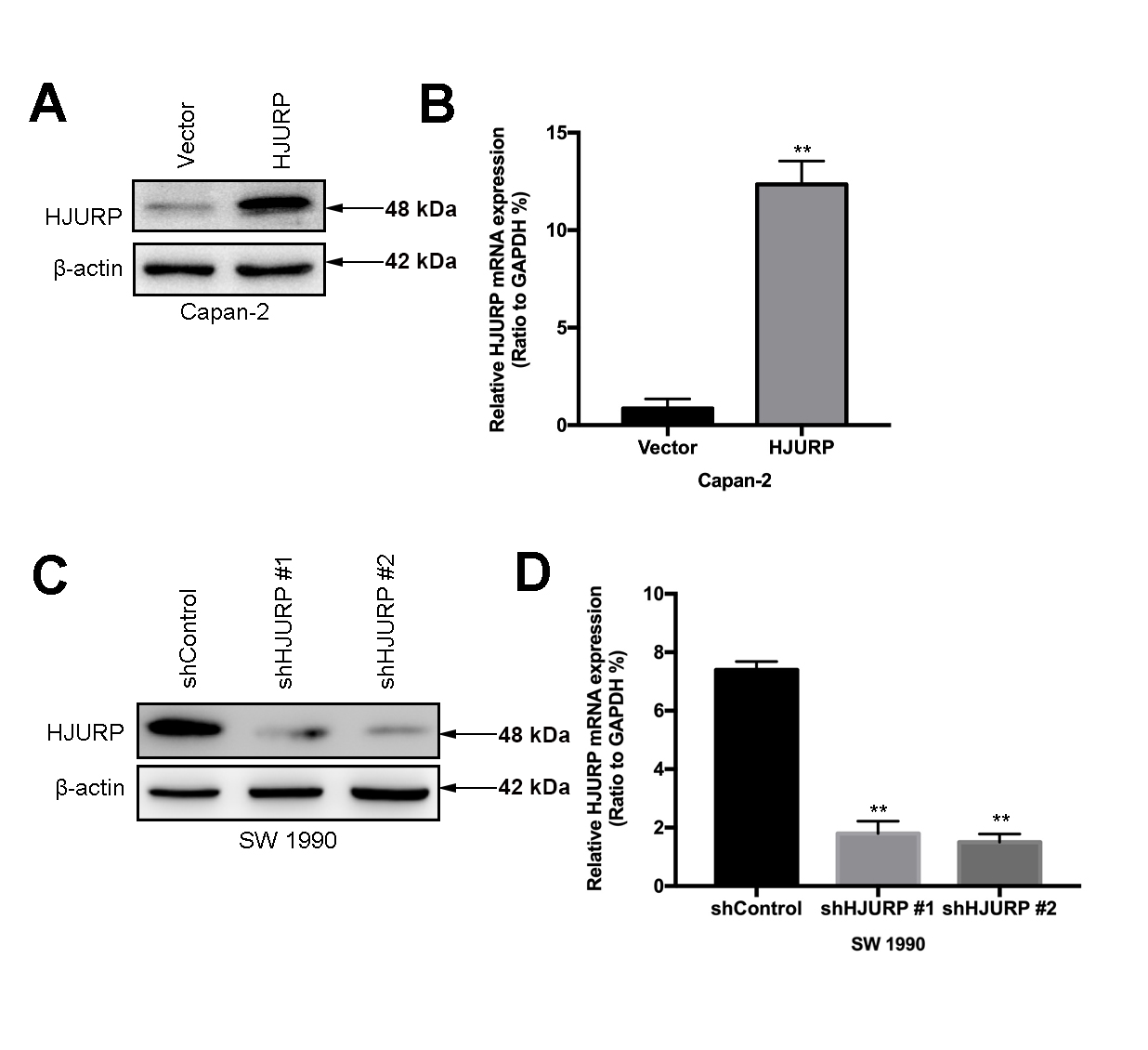

Supplement: Supplementary file 2 — Supplemental Fig. 2 [file 41419_2020_2595_MOESM2_ESM.tif]

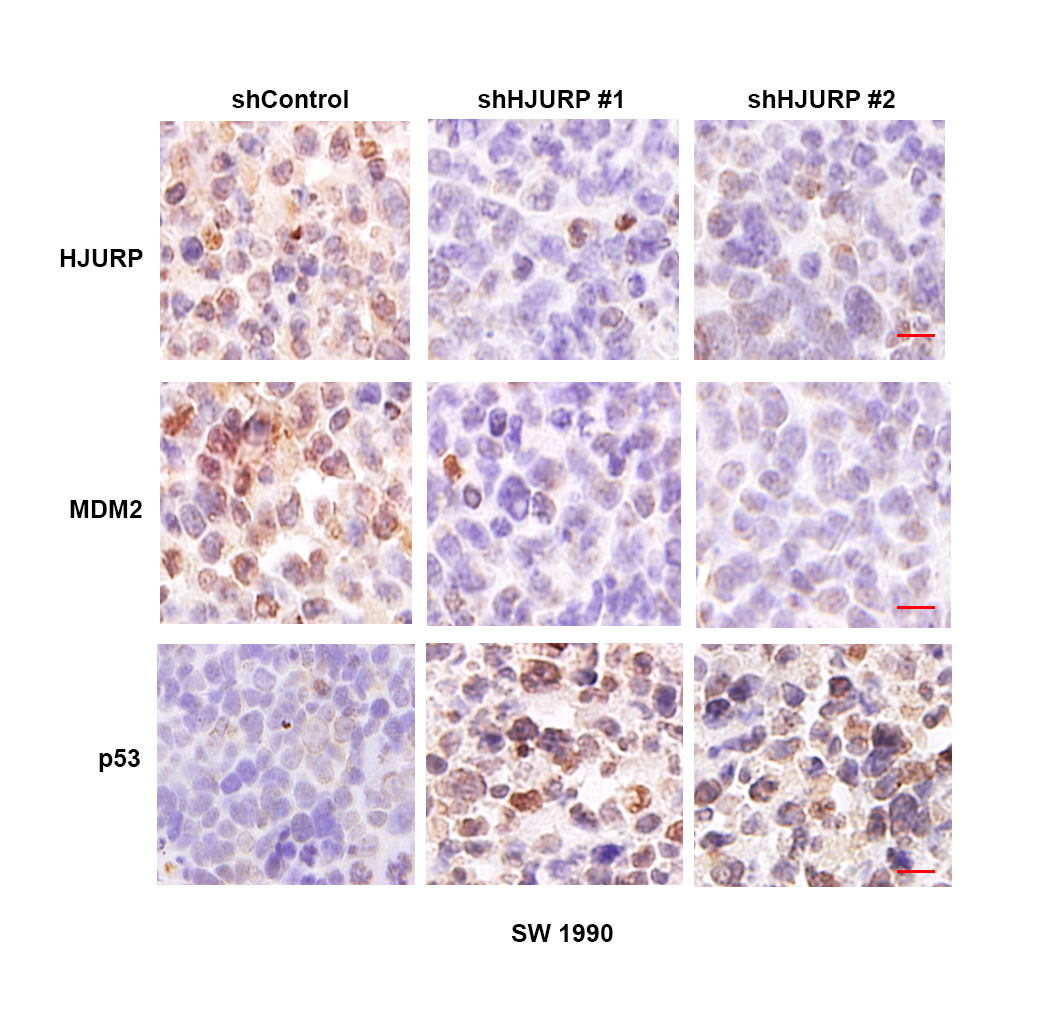

Supplement: Supplementary file 3 — Supplemental Fig. 3 [file 41419_2020_2595_MOESM3_ESM.tif]

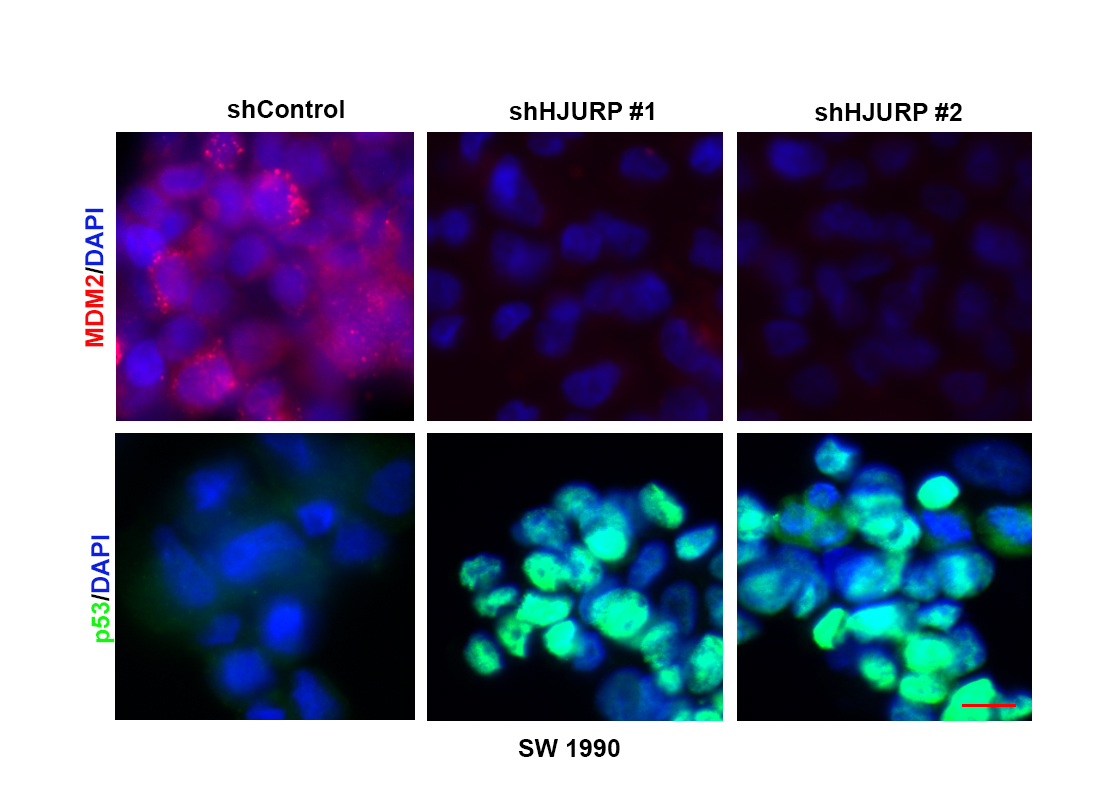

Supplement: Supplementary file 4 — Supplemental Fig. 4 [file 41419_2020_2595_MOESM4_ESM.tif]

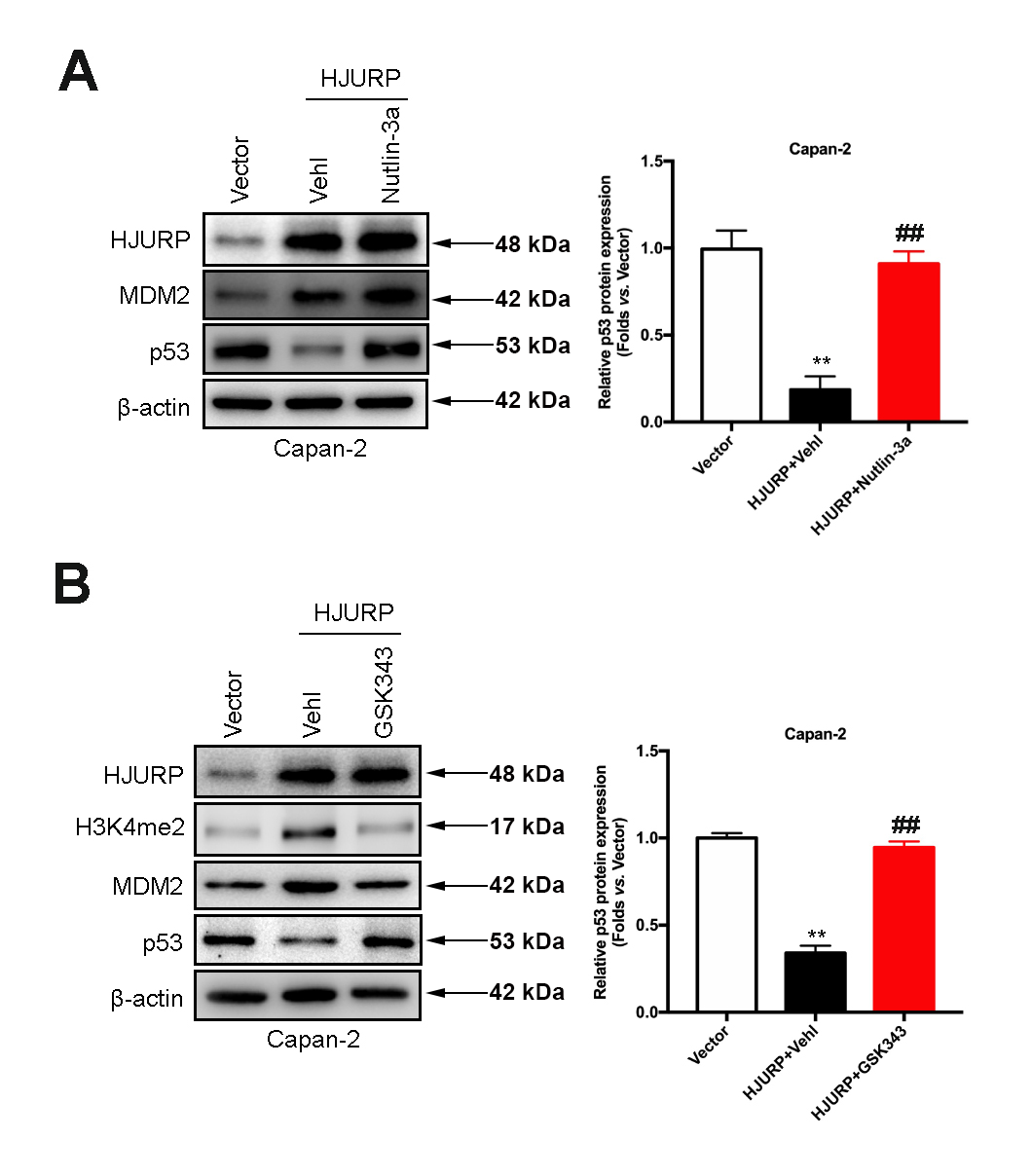

Supplement: Supplementary file 5 — Supplemental Fig. 5 [file 41419_2020_2595_MOESM5_ESM.tif]
