# Supplementary material for: Holliday junction recognition protein promotes pancreatic cancer growth and metastasis via modulation of the MDM2/p53 signaling
Source: Cell Death Dis. 2020 May 21;11(5):386. doi: 10.1038/s41419-020-2595-9 (PMC7242411; doi:10.1038/s41419-020-2595-9)
Supplement: Supplementary file 6 — Supplemental Figure legends [file 41419_2020_2595_MOESM6_ESM.docx]

**Supplemental Figure legends**

**sFig. 1** ROC curve analysis of HJURP in pancreatic cancer.

**sFig. 2** Transfection efficiency of transformed Capan-2 and SW 1990 cells described

in Fig.3. (a-f). Immunoblotting (a, c), and RT-qPCR (b, d) to measure transfection efficiency of Capan-2 (a-c) and SW 1990 cells (d-f) described in Fig.3. All experiments were repeated at least three times, and representative data are shown. Data are means ±SEM. **p < 0.01.

**sFig. 3** Representative images of IHC staining of MDM2 and p53 in transformed Capan-2 cells. Scale bars, 20 μm.

**sFig. 4** Representative images of immunofluorescence of MDM2 and p53 in transformed SW 1990 cells. Scale bars, 20 μm.

**sFig. 5** (a, b) Capan-2 cells were treated with Nutlin-3a, and p53 and MDM2 expression level was detected by immunoblotting. (c, d) Capan-2 cells were treated with GSK343, and H3K4me2, p53 and MDM2 expression level was detected by immunoblotting. Data are means ±SEM. **p < 0.01.
